# Mechanisms of Cardiovascular Calcification and Experimental Models: Impact of Vitamin K Antagonists

**DOI:** 10.3390/jcm13051405

**Published:** 2024-02-29

**Authors:** Chiara Siracusa, Annarita Carino, Nicole Carabetta, Marzia Manica, Jolanda Sabatino, Eleonora Cianflone, Isabella Leo, Antonio Strangio, Daniele Torella, Salvatore De Rosa

**Affiliations:** 1 Department of Medical and Surgical Sciences, Magna Graecia University, 88100 Catanzaro, Italy; chiara.siracusa@unicz.it (C.S.); annacarino@unicz.it (A.C.); nicole.carabetta95@gmail.com (N.C.); marzia.manica@studenti.unicz.it (M.M.); cianflone@unicz.it (E.C.); 2 Department of Experimental and Clinical Medicine, Magna Graecia University, 88100 Catanzaro, Italy; sabatino@unicz.it (J.S.); i.leo@unicz.it (I.L.); antonio91strangio@gmail.com (A.S.); dtorella@unicz.it (D.T.)

**Keywords:** calcification, atherosclerosis, anticoagulation, vitamin K

## Abstract

Cardiovascular calcification is a multifactorial and complex process involving an array of molecular mechanisms eventually leading to calcium deposition within the arterial walls. This process increases arterial stiffness, decreases elasticity, influences shear stress events and is related to an increased risk of morbidity and mortality associated with cardiovascular disease. In numerous in vivo and in vitro models, warfarin therapy has been shown to cause vascular calcification in the arterial wall. However, the exact mechanisms of calcification formation with warfarin remain largely unknown, although several molecular pathways have been identified. Circulating miRNA have been evaluated as biomarkers for a wide range of cardiovascular diseases, but their exact role in cardiovascular calcification is limited. This review aims to describe the current state-of-the-art research on the impact of warfarin treatment on the development of vascular calcification and to highlight potential molecular targets, including microRNA, within the implicated pathways.

## 1. Introduction

Vascular calcification (VC) represents a complex biological phenomenon characterized by calcium phosphate deposition within vascular structures [1]. This condition is frequently observed in various cardiovascular and medical conditions, particularly chronic kidney disease and diabetes [2]. The pathogenesis of VC is not only related to an increased availability of phosphorous and calcium, but involves a series of intricate, well-coordinated biological pathways, including dysregulation of osteochondrogenic processes and perturbation of signaling pathways that ordinarily inhibit calcium deposition [2]. It has been in fact demonstrated that dysfunctional calcium homeostasis is a prerequisite for the development of VC [2]. In addition to traditionally established osteogenic signaling, loss of defensive mechanisms by micro organelle dysfunction, including hyper-fragmented mitochondria, increased mitochondrial oxidative stress, defective autophagy or mitophagy, and endoplasmic reticulum (ER) stress, may all contribute to VC [2]. Interestingly, VC can involve both the intimal and medial part of a blood vessel, each with different clinical and pathological implications. Medial calcifications are primarily linked with conditions such as kidney disease (CKD), diabetes mellitus (T2DM), hypertension, and aging, while intimal calcifications correlate with arterial occlusions and thrombotic events [3]. Inflammatory cells, including macrophages, play a pivotal role in fostering vascular calcification through the release of pro-inflammatory cytokines such as TNF-a, IL-1β, and IL-6 [4], which collectively contribute to the calcific milieu within the vascular system.

Vascular calcifications have emerged as a critical prognostic indicator for cardiovascular incidents [5]. An increased coronary artery calcium score (CAC) correlates in fact with the severity of coronary artery disease (CAD) [6] and an increased rate of CAC progression correlates with adverse cardiac events [7]. Traditionally considered as an aging by product, VC is now recognized as an actively regulated process akin to bone mineralization, serving as both a cardiovascular risk marker and a treatment target, with ongoing development of detection methods to enhance its clinical utility [8,9,10].

Therefore, to devise effective vasculo-protective treatments, a comprehensive understanding of the mechanisms behind VC development is imperative.

## 2. Arterial Calcification

**Figure 1:**
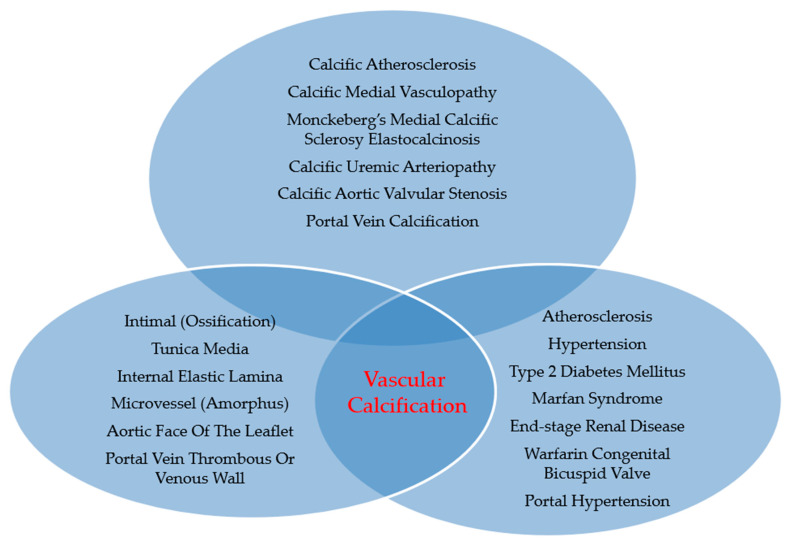
Types, locations, features, and associated condition(s) of VC.

Inducing osteogenic differentiation in a subset of vascular cells is a fundamental biological process in atherosclerotic calcification, a very common form of vascular disease. The evolution of this process is regulated by specific inflammatory molecules, namely lipoproteins and cytokines, normally present in the atheromatous components of plaques [11,12]. Figure 1 describes the types, locations, and features of vascular calcification, along with associated clinical conditions.

Arterial calcifications are sub-classified according to histo-anatomical and ethiological criteria. Histologically, they are divided into osteomorphic, chondromorphic, or amorphic. Etiologically, they can instead be further sub-categorized as metastatic and dystrophic arterial calcification [13].

Clinical studies have already established a connection between the presence of dyslipidaemia and the onset, progression, and severity of VC [14,15,16]. On a pathophysiological level, it is known that statins prevent calcification in hyperlipidaemic mice through the Gas-6/Axl signaling [17,18,19], even though these results were not paralleled in randomized clinical trials [17]. An osteogenic signaling mechanism is present in intimal atherosclerotic calcification [20,21]^s^. Both type 2 bone morphogenetic protein (BMP-2) and osteopontin have been found in human atherosclerotic plaque, where they have been recognized as hallmarks of calcification and inflammatory destabilization of the plaque [22,23,24,25]. Oxidative stress promotes vascular cell calcification in vitro, effectively inhibited by the use of antioxidant factors such as ω-3 fatty acid and high-density lipoproteins [26].

Murine models of atherosclerotic calcification have been developed to further understand the underlying mechanism involved in their development. Spontaneous vascular calcification has been founding several genetically different inbred mouse strains, including C5BL/6, Balb/C, C3H/HeJ, DBA/2J, SM/J, and MRL-lpr/lpr mice, and found to increase following a high-fat/high-cholesterol diet [26].

Arterial VC development was not the same among the different strains, indicating that some genetic component may have influenced the phenotype. Vascular calcification has been observed, for instance, in apoE-deficient mice, and is accelerated by osteopontin deficit [27]. The Klotho mouse also tends to develop calcification along with atherosclerosis and calcification, with calcifications located in the media and sensitive to phosphatemia [28].

Calcified vascular cells have many features of microvascular pericytes and are distinguished from traditional SMCs due to the presence of a specific superficial ganglioside. Unlike skeletal osteoblast cultures that stay in a monolayer, require ascorbate and phosphate supplements, and mineralize more uniformly, these cells naturally generate minerals and tend to form multicellular nodules that bear a histological resemblance to atherosclerotic plaque and nodules, found in the aortic valve [28]. The analysis of these nodules allowed us to unravel an underlying mechanism, the so-called ‘diffusion reaction’ process, controlled by the interaction between BMP-2 and its inhibitors, specifically the Matrix Gla Protein (MGP). A computational model of these mechanisms has successfully predicted that warfarin, an inhibitor of MGP, would double the spatial frequency of the calcium [29].

## 3. Medial Artery Calcification

Medial artery calcification is a typical feature of T2DM and chronic kidney disease. Although initially considered a benign feature and not associated with stenosis or intraluminal thrombosis, medial artery calcification is now a recognized malignant feature, linked with increased cardiovascular mortality and higher risk of amputation in patients with both type 2 diabetes mellitus and end-stage kidney disease. The mechanisms involved in the development of medial calcification are heterogeneous and vary in regard the different etiologies [30,31,32]; for instance, diabetic arterial calcification is predominantly made of hydroxyapatite, whereas whitlockite seems to be the main component in case of vitamin D toxicity [33].

In the context of T2DM, the BMP-2/Mxs2/Wnt signaling, linked to inflammatory redox status, plays a key role in the early stages of medial calcification, in a pathway that appears to be independent from Runx-2/Cbfa1. Another key element is the elastin degradation, an early feature in many forms of medial calcification. The resulting elastin metabolites can, in fact, activate and even nucleate calcium deposition [34].

Matrix metalloproteinase-9, an elastase expressed at the vascular level after damage, facilitates medial calcium deposition in models using warfarin/vitamin K [29,35].

## 4. Calcification of Cardiac Valves

Aortic valve calcification, particularly common in elderly populations and patients with heart failure or end-stage renal disease, is associated with significant mortality [36]. Mechanical stress, alongside metabolic and inflammatory disruptions, is a contributing factor. Advances in understanding the cellular mechanisms of calcific aortic stenosis have been made through the study of calcifying cells from aortic valve tissues. Notably, a study of aortic valve specimens highlighted inflammation, bone morphogenetic protein (BMP) expression, and the presence of mature bone tissue in over 10% of cases [37]. Active osteochondrogenic differentiation and signaling has been observed in human valves and animal models. This includes the differential expression of Wnt-3 and the LDLR-related protein (LRP5)-dependent activation of the Wnt signaling cascade, which leads to β-catenin accumulation in the nucleus. The latter serves as a critical co-factor for LEF1/TCF7 and Smad transcription [38]. Why active osteogenic processes are more commonly observed in aortic cusps or the mitral annulus, whereas they are virtually absent in mitral valve leaflets, still remains unclear, although an influence of embryonic development or mechanical stress may be implicated [39].

## 5. Calcific Uremic Arteriolopathy

Calcific uremic arteriolopathy (CUA), or calciphylaxis, is a rare and serious condition caused by calcium accumulation within small blood vessels of adipose and skin tissues [40]. This can eventually lead to intraluminal thrombosis, subsequent ischaemia, painful skin ulcers, and life-threatening infections [40,41].

Although the exact cause of calciphylaxis is unknown, abnormalities of factors involved in the coagulation cascade and dysregulation of calcium metabolism may be involved.

Risk factors for calciphylaxis include female sex, obesity, diabetes, coagulation abnormalities, long-standing chronic kidney disease, particularly in dialysis, previous kidney transplantation, dysregulation of calcium and phosphorus homeostasis, and the use of certain medications such as warfarin, calcium-binding agents or corticosteroids [42]. Calciphylaxis clinically manifests as large purple net-like skin lesions, and/or very painful lumps that ulcerate, creating open sores with a black-brown crust that fail to heal, more frequently involving skin areas with high fat content (i.e., the stomach and thigh). The treatment focuses on managing symptoms and includes intensive wound care. In addition, drugs such as sodium thiosulfate (chelating agent that stabilize calcium and iron) and Cinacalcet (a calcimimetic agent controlling parathyroid hormone levels) [41] can be also used in these patients, especially in patients with a history of long-standing treatment with warfarin [43].

## 6. Molecular Mechanisms Underlying Vascular Calcifications Associated with VKA

**Figure 2:**
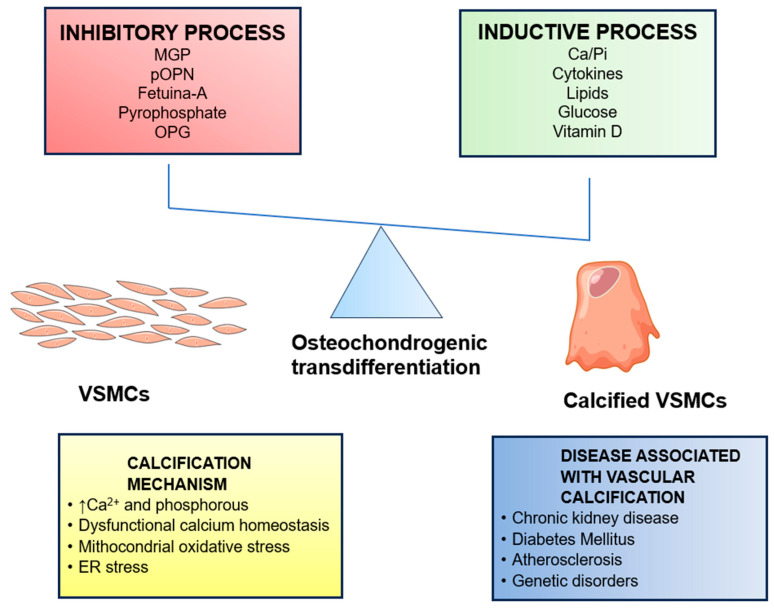
The several factors involved in VC development.

Several factors contribute to VC development, including circulating nucleation complexes/paracrine factors usually involved in bone remodeling, abnormal calcium and phosphate homeostasis, inflammation, matrix degradation with elastolysis, failure of physiologic anti-calcific processes or loss of specific inhibitors of calcification (cMGP, pOPN, fetuin A, pyrophosphate), defective osteoclastogenesis, osteochondrogenic differentiation of vascular cells, uremic serum, and chronic use of vitamin K antagonists [28]. The factors involved in VC development are illustrated in Figure 2.

Warfarin and other vitamin K antagonists (VKAs) can be prescribed to patients with indication for chronic anticoagulation therapy, including atrial fibrillation, stroke, and hyper-coagulable disorders. Their anticoagulant effects depend on the interference with the peripheral production of vitamin K-dependent proteins [18].

To understand the cellular and molecular mechanisms subtended to vascular calcification, various in vitro and animal models have been developed [32,44,45,46]. There is ongoing debate about the types of calcifying cells in the vasculature, which include vascular smooth muscle cells (VSMCs) in both contractile and proliferative synthetic phenotypes, calcifying vascular cells (CVCs), pericytes, mesenchymal stem cells (both circulating and resident), adventitial cells, myofibroblasts, fibrocytes (circulating), and calcifying vascular progenitor cells (both circulating and resident) [47].

Gene deletion in mice is a useful technique to determine the role of a specific protein in some pathophysiological processes. For instance, the importance of the phosphaturic hormone fibroblast growth factor 23 (FGF23) along with its co-factor Klotho have been demonstrated in the process of vascular calcification: both FGF23- and Klotho-deficient mice exhibit extensive vascular and soft tissue calcifications [48].

**Figure 3:**
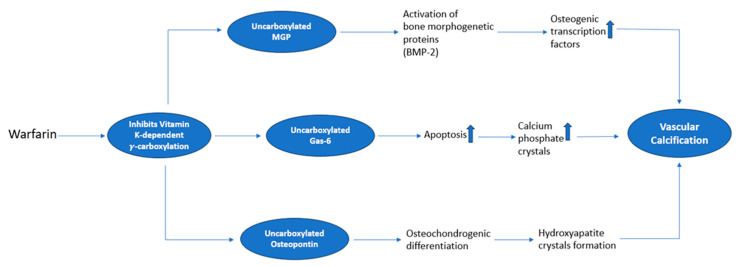
Molecular mechanisms of warfarin in VC.

Warfarin is able to inhibit MGP and other γ-carboxylated proteins regulating mineralization, including osteocalcin and Gas-6 [29]. The mechanisms by which warfarin can induce vascular calcification are illustrated in Figure 3. Thereby, warfarin and other VKAs can possibly interfere with calcification, both affecting fetuin A and BMP-2 [28]. In fact, MGP requires posttranslational modification by γ-carboxylation to become active. A high dose of vitamin K, key for ensuring γ-carboxylation, is able to revert warfarin-induced vascular calcium deposition in animal models [29]. Several murine knockout models of genes involved in osteogenesis have provided new insights into the pathogenesis of arterial calcification. MGP, osteoprotegerin (OPG) [49], and fetuin A knockout mice display extensive aortic calcifications, hence proving the preventive role of these proteins in the calcification process [2]. The administration of different stimulants such as indoxyl sulfate [50], vitamin D [51], nicotine [52], and warfarin [53] can induce vascular calcification in rodents.

A summary of the current state-of-the-art knowledge about in vitro and in vivo studies related to warfarin treatment and its implications in cardiovascular events is shown in Table 1 and Table 2.

The molecular mechanisms of warfarin action remain largely unknown. Numerous studies have indicated that warfarin may induce vascular calcification in arteries, but the specific molecular level interactions are not well-documented in the literature. Our analysis of various papers and viewpoints suggests that it is challenging to construct a clear model of action for this molecule.

Warfarin, a commonly used anticoagulant, appears to interfere with the function of MGP and may be involved in the dysregulation of fetuin A, osteopontin expression, pyrophosphate, and other molecular inhibitors. It may also be associated with altered mechanisms of balance between Ca/Pi and hormonal disorders, interacting with estradiol, insulin, or vitamin D3 [70].

The use of warfarin in patients with significant calcifications, who are likely to have inefficient inhibitory ability, may further worsen calcification. The association of warfarin treatment with calciphylaxis supports this hypothesis. Genetic factors, such as polymorphisms that could potentially affect genes inhibiting warfarin functionality, may also have a relevant role in this context [18].

Interactions with other drugs taken concurrently with warfarin can alter its efficiency and, over time, cause damage to the arterial wall. Additionally, specific circulating microRNAs in the blood could potentially upregulate or downregulate warfarin effects under certain hemodynamic conditions.

Oxidative mechanisms and specific molecular targets have been shown to mediate warfarin action and decrease vascular calcification in vitro [66,67,68,69]. Many studies have analyzed targets such as beta-catenin and ets-1 [58]. A link to bone formation and processes involving bone mineralization and crystals, such as hydroxyapatite, should not be excluded from potential interactions with warfarin [71].

There is a strong correlation between the presence of vascular calcification and cardiovascular disease, particularly with adverse outcome such as ischemic heart disease and cardiovascular death [72].

## 7. miRNA in Cardiovascular Calcification

A recent study has unveiled the first miRNA-dependent mechanism responsible for the progression of vascular calcification. Dysregulation of miR-125b has been proven to contribute to the transformation of human coronary arterial SMCs into osteoblast-like cells, a process partially occurring through the targeting of the transcription factor osterix [73]. Observations from in vivo animal studies suggest that miR125b levels are reduced in the presence of aortic calcification of apolipoprotein-deficient (Apoe) mice [73]. In addition, the miRNA-processing enzymes DROSHA and DICER, essential for SMC function, were reduced in calcified SMCs [74]. Another mi-RNA, MiR-204, an inhibitor of osteoblastogenesis [75] interacting with the Runx2-miRNA-cluster in osteoblasts [76], was found to contribute to SMC calcification in vitro and in vivo [34,77]. In detail, in vitro studies have demonstrated its role as a suppressor of SMC calcification, as it directly targets the 3′ untranslated region of the Runx2 gene in mouse SMCs. Similarly, in a mouse model of vitamin D3-induced vascular calcification, the overexpression of miR-204 through agomiRs reduced medial calcification and Runx2 expression, mostly to control levels. Changes in the expression level of miR-143/145 cluster promote differentiation and, at the same time, inhibit proliferation of SMCs correlating with the onset of vascular calcification [78]. When human primary SMCs are exposed to pathophysiological levels of phosphate, the expression of miR-143 and miR-145 decreases. While a direct functional role of miR-143 and miR-145 in vascular calcification has not been established in some studies, this theory is backed by findings in literature related to diseases associated with calcification. In fact, miR-145 is known to promote SMC differentiation by targeting the Kruppel-like factor (KLF)-4; in turn, KLF4 mediates high phosphate-induced transition of SMCs into osteogenic cells. In line with these findings, miR-145 was associated with the microcalcification of atherosclerotic plaques in patients [79]. Many other miRNAs are involved in vascular calcification; in particular, we assembled different miRNAs that increase or decrease their level of expression, as reported in the literature, and investigated them for CAD, aortic stenosis, and arteriosclerosis obliterans (AO). MiR-21, miR-34a, miR-146a, miR-146b-5p, and miR-210 are increased in atherosclerotic arteries [80]. MiR-105, miR-520b, and miR-30bn are decreased in the atherosclerotic carotid artery. Increased levels of miR-22, miR-27a, miR-141, miR-124, miR-125b, miR-185, miR-187, miR-194, miR-211, miR-330, miR-370, miR-449, miR-486, miR-551, miR-564, miR-575, and miR-585 were reported in the bicuspid aortic valve [81], a condition that is frequently associated with calcification of the aortic cusps. On the contrary, expression levels of miR-622, miR-637, miR-648, miR-1202, miR-1282, miR-1469, miR-1908, miR-1972, miR-30e, miR-32, miR-145, miR-151, miR-152, miR-190, miR-373, and miR-768 are increased in the same condition [81]. In addition, flow-responsive miRNAs are associated with the calcific degeneration of both the tricuspid and bicuspid aortic valves [51]. The observation that clusters of miRNAs regulate cellular and tissue balance suggests a connection between natural bone development and vascular calcification. Future research could elucidate the relationship between known osteogenesis signaling pathways and the pathological outcomes they lead to, paving the way for a more profound understanding of the cellular processes behind calcification.

Circulating miRNAs are also biomarkers found in conditions associated with vascular calcification, such as CKD, T2DM, and CAD. These miRNAs likely act by affecting blood vessels directly. Among others, miR-21 seems to have a key role in atherosclerosis progression and is expressed at higher levels in atherosclerotic arteries and in the sclerotic intima of the lower-extremity vessels compared to healthy vessels. However, circulating levels of miR-21 in atherosclerosis (serum), T2DM, or CKD (plasma) are reduced [82]. The reason for this discrepancy is not fully understood and warrants further investigation. It is hypothesized that the uptake of miRNAs packaged inside exosomes or apoptotic bodies may be increased in injured tissues, with subsequent lowering of circulating miRNAs. The expression levels of miR-146a measured in peripheral blood mononuclear cells is increased in CAD patients. Additionally, miR-155, a miRNA associated with pro-inflammatory processes, is decreased in the serum of CAD and plasma of CKD patients. Deficiency of miR-155 fosters atherosclerotic plaque development and decreases plaque stability, suggesting that it acts as an anti-inflammatory and athero-protective miRNA; furthermore, MiR-155 targets the angiotensin II receptor [83]. To validate the hypothesis that the renin-angiotensin system is involved in VC, it has been shown that blocking angiotensin receptors can prevent arterial calcification by interfering with the process of vascular bone formation in living organisms.

Circulating miRNAs associated with vascular calcification in CAD are illustrated in Table 3.

## 8. miRNA Transport in Vesicles and Calcification

The exact mechanism through which miRNAs are delivered to target cells in an active state is not well understood. In normal conditions, it is believed that extracellular vesicles may attach to membrane proteins on the surface of target cells via receptor–ligand interactions, which may activate genetic cellular pathways. These vesicles can connect with cell surface receptors and enter the cell through endocytosis. After entering the cell, they may merge with the cell membrane, releasing their cargo into the cell’s cytoplasm. This process enables the contents of the vesicles to interact directly with the cell’s internal components [84].

Extracellular vesicles may also have a role in matrix mineralization. The release of vesicles from SMCs is in fact a key event in the initiation and promotion of SMC calcification. When SMCs are exposed to high calcium levels, there is an increase in the formation of calcifying matrix vesicles, acting as early nucleation sites for calcification, and the loss of fetuin-A, an inhibitor of mineral nucleation. Hydroxyapatite nanocrystals shed from these vesicles may further promote mineralization through direct effects on the SMC phenotype [81].

In a pro-osteogenic environment, the vesicle-mediated transport of miRNA in the vasculature may be disrupted through differential miRNA packaging into the vesicle, decreased miRNA stability due to increased exonuclease activity, increased vesicle degradation, or blocked or non-specific uptake of vesicles into the target cell due to mineral nucleation on the outer membrane [81].

In addition, microcalcifications can directly contribute to plaque rupture, leading to thrombosis [84,85].

Experimental evidence suggests that exosomal miRNAs are involved in the development of VC [86,87,88]. For instance, increased levels of miR-135, miR-762, miR-714, and miR-712 in VSMCs were shown to contribute to VC by interfering with the calcium efflux protein [89]. Additionally, the positive feedback loop of Runx2/miR-3960/miR-2861 is known to play a role in the conversion of VSMCs into osteoblasts, thereby promoting VC formation [90]. In addition, miR-324-3p was shown to regulate calcification via IGF1R, PI3KCA, and MAP2K1 in rat smooth muscle and the MOVAS-1 cell line. In this model, the levels of exosomal let-7e-5p decreased while exosomal miR-324-3p levels increased [91]. Hence, engineered exosomes could potentially be developed into a new generation of targeted specific microRNAs or small RNA-interfering molecules to prevent VC and enhance the stability of atherosclerotic plaques.

## 9. Conclusions

Vascular calcification is an active process, thereby mediated by specific mechanisms in response to an array of stimuli as well as to a number of modulating factors. VC is a frequent effect of aging, and it is linked to multiple disorders, including T2DM, CKD, and cardiovascular disease. Therefore, deposition of calcium phosphate can occur in cardiac valves or in the arterial walls. In this context, VKAs appear to be involved in VC, through mechanisms that include MGP inhibition and the γ-carboxylation of other proteins which regulate mineralization, such as Gas-5 and Osteocalcin. Furthermore, experimental results support a possible involvement of specific miRNAs (miR-145, miR-29, and others) in the fine tuning of the processes underlying vascular calcifications. The multiple experimental findings that followed the understanding of the impact exerted by VKAs on the calcification of soft tissue has largely contributed to improve our understanding of the biological mechanisms responsible for the development of vascular calcifications and to disentangle some of the regulating processes. These results open the way to their potential exploitation to develop vasculo-protective therapies, especially in elderly patients or those with chronic kidney disease and on hemodialysis.

## Figures and Tables

**Table 1:** In vivo studies related to warfarin treatment and its pathogenetic role in cardiovascular events.

Authors	Outcome Animal Models In Vivo (Rats)
Price P.A. et al., 1998 [29]	This study explores the role of MGP in preventing calcification of arteries and heart valves following treatment with warfarin and vitamin K.
Price P.A. et al., 2000 [33]	Growth and vitamin D treatment enhance the extent of artery calcification in rats given sufficient doses of Warfarin to inhibit γ-carboxylation of MGP.
Price P.A. et al., 2001 [14]	Osteoprotegerin can potently inhibit the calcification of arteries induced by warfarin and vitamin D.
Price P.A. et al., 2001 [54]	Bisphosphonates inhibit the calcification of arteries and heart valves at doses comparable to the doses that inhibit bone resorption.
Price P.A. et al., 2006 [55]	The elastic lamellae of devitalized aortas calcify rapidly in serum.
Price P.A. et al., 2006 [56]	Medial artery calcification in uremic rats correlates with increased serum bone Gla protein (BGP; osteocalcin).
Essalihi et al., 2005 [57]	Vascular mineral loss induced by the blockade of endothelin receptors seems dependent on the activation of membrane-bound CA IV.
Neradova et al., 2022 [19]	Treatment of phosphate chelators with a high vitamin K2 content prevents vitamin K deficiency and attenuates the development of VC.
Van den Bergh et al., 2021 [21]	Endothelial cells could potentially function as an additional source of osteogenic progenitor cells in arterial calcification.
De Maré et al., 2022 [45]	Sclerostin induces a protective effect during vascular calcification in murine models of VC induced by renal failure or inhibition of MGP.
Van den Bergh et al., 2022 [20]	Endothelial dysfunction can contribute to the early stages of development of medial artery calcification.
Opdebeeck et al., 2023 [30]	Inhibition of TNAP SBI-425 attenuated medial artery calcification in a rat model induced by warfarin.
Uto et al., 2021 [31]	The inhibition of lysyl oxidase (LOX) by β-aminopropionitrile (BAPN) attenuated medial artery calcification in rat models fed a diet containing warfarin and vitamin K1.
Fang et al., 2020 [44]	The mir-29b/TGF-β3 axis could play a regulatory role in the pathogenesis of VC.
Opdebeeck et al., 2020 [32]	TNAP SBI425 inhibitor significantly reduced aortic and arterial calcification in warfarin-induced VC models.
De Maré et al., 2019 [46]	The production of sclerostin in serum, aorta, and bone was investigated in rats with warfarin-induced VC.

**Table 2:** In vitro studies related to warfarin treatment and its pathogenetic role in cardiovascular events.

Authors	Outcome Models In Vitro (Cell Culture)
Beazley K.E. et al., 2012 [58]	Inhibition of canonical β-catenin pathway or TG2 activity prevents warfarin-regulated calcification.
Beazley K.E. et al., 2013 [38]	New β-catenin-targeting strategies prevent VC induced by warfarin and identify quercetin as a potential therapeutic.
Beazley K.E. et al., 2013 [59]	Inhibition of the TG2/β-catenin signaling axis seems to prevent warfarin-induced elastocalcinosis and to control isolated systolic hypertension.
Zhiyu H. et al., 2003 [60]	Adrenomedullin and PTHrP inhibited VSMC calcification partially through the cAMP/PKA pathway, whereas CNP inhibited VSMC calcification through the cGMP/PKG pathway.
Shanahan C Met. et al., 1998 [61]	Several other Gla-containing proteins with the potential to regulate or perhaps contribute to VC are present in the human vasculature.
Sheng Ying Wu et al., 2003 [62]	Endothelin might be involved in the pathogenesis of vascular calcification.
Trion A. et al., 2004 [26]	VSMCs contribute to the development of an atherosclerotic lesion by migration, proliferation, and secretion of matrix components.
Liu Y. et al., 2013 [63]	Prelamin A promotes VSMC calcification and aging by inducing persistent DNA damage signaling, which acts upstream of VSMC osteogenic differentiation and the senescence-associated secretory phenotype.
Son B.K. et al., 2012 [64]	TM is a novel molecule that promotes apoptosis and vascular calcification by regulation of Gas6, presumably via EGF receptors/ERK axes.
Rangrez AY et al., 2012 [65]	Results suggest that (i) high levels of Pi increase VSMC migration and calcification, (ii) altered expression levels of miR-223 could play a part in this process, and (iii) miR-223 is a potential new biomarker of VSMC damage.
Nie et al., 2019 [66]	Activation of the Wnt/β-catenin pathway regulates arterial calcification by activation of the OPG/RANKL system.
Seime et al., 2021 [67]	Proteoglycan 4 (PRG4) modulates the function of SMC and the osteogenic phenotype during vascular remodeling and intimal calcification.
Liu et al., 2021 [68]	EGB761 inhibited vascular calcification and osteogenic differentiation by suppressing the BMP2/Smad1/5/Runx2 signaling pathway.
Wei et al., 2020 [69]	Warfarin can induce senescence of vascular cells and contribute to the spread of vascular inflammation and oxidative stress via SASP.

**Table 3:** Circulating miRNAs in coronary artery disease associated with vascular calcification.

Coronary Artery Disease	Source	Finding
miR-133a, miR-208a, miR-146a/b, miR34a, miR-221, miR-222, miR-122, miR-370, miR-624	Serum, peripheral blood mononuclear cells, plasma, and platelets	Level of expression: increased
miR-17, miR-21, miR-20a, miR92a, miR-27a, miR-22a, miR-126, miR-145, miR-155, miR221, miR-130a, miR208b, let-7d, miR-135a, miR147, let-7i, miR-140, miR-182, miR-181a	Serum, endothelial progenitor cell, peripheral blood mononuclear cell, monocytes	Level of expression: decreased
